# Leaf Disc Assays for Rapid Measurement of Antioxidant Activity

**DOI:** 10.1038/s41598-018-38036-x

**Published:** 2019-02-13

**Authors:** Deepak M. Kasote, Guddadarangavvanahally K. Jayaprakasha, Bhimanagouda S. Patil

**Affiliations:** Vegetable and Fruit Improvement Center, Department of Horticultural Sciences, Texas A&M University, 1500 Research Parkway, A120, College Station, TX 77845-2119 USA

## Abstract

Antioxidant levels are key parameters for studies of food quality, stress responses, and plant health. Herein, we have demonstrated that excised leaf disc has both radical scavenging activity and reducing power, and used this concept to develop 2,2-diphenyl-1-picrylhydrazyl (DPPH), 2,2′-azino-bis(3-ethylbenzothiazoline-6-sulfonic acid) (ABTS), and potassium permanganate reduction (PPR) leaf disc assays. Reaction time and reagent concentration for these assays were optimized using leaves from spinach, kale, collards, mustard, and watermelon. Further, these assays were validated for linearity and intra-assay precision. Ultra-high performance liquid chromatography coupled to an electrospray quadrupole time-of-flight mass spectrometer (UPLC/ESI-HR-QTOFMS) was used for phytochemical profiling and studying relative abundances of certain phenolic compounds in various leaf discs suspended and cell-free extracts. The mass spectral analysis showed that leaf disc suspended methanolic extracts had almost same phytochemical profiles to those of cell-free extracts. The DPPH leaf disc assay demonstrated better radical scavenging potential than the conventional cell-free extract method. By contrast, the observed antioxidant activity values in ABTS and PPR leaf disc assays were lower than those of conventional cell-free extract-based methods. In conclusion, the developed leaf disc assays are simple and rapid for the qualitative and comparative assessment of the antioxidant potential of leaf samples, as well as can be a good alternative to conventional cell-free extract based methods.

## Introduction

Plants are constantly experiencing oxidative stress due to several abiotic and biotic factors. Consequently, they have an inherited efficient complex response network of enzymatic and non-enzymatic antioxidants to prevent deleterious effect reactive oxygen species such as hydrogen peroxide, superoxide and hydroxyl radicals, which are normally produced in excess upon exposure to biotic and abiotic stresses^1,2^. The enzymatic system mainly includes superoxide dismutase, catalase, glutathione peroxidase, and glutathione reductase whereas, the non-enzymatic system consists of low and high molecular weight phyto-antioxidants such as ascorbic acid, phenolics, and flavonoids^3,4^. Non-enzymatic phyto-antioxidants play a vital role in the plant growth, defense response, and development, together with their main cellular protective function against oxidative stress^5,6^. Due to this reason, researchers are trying to enhance the biosynthesis and accumulation of phyto-antioxidants in plants to provide abiotic and biotic stress tolerance using genetic engineering, plant breeding, and seed priming strategies^7–9^.

In general, the antioxidant potential of the plant samples is routinely assessed by three approaches such as (a) direct measurement of antioxidant enzymes activity, (b) *in vitro* radical scavenging and reducing power assessment and (c) measuring the protective response of plant samples against chemical-induced oxidative stress. However, each of these approaches has its own limitations about applicability^3^. Several *in vitro* radical scavenging and reducing power assessment assays such as 2,2-diphenyl-1-picrylhydrazyl (DPPH), 2,2′-azino-bis(3-ethylbenzothiazoline-6-sulfonic acid (ABTS) and ferric reducing antioxidant power (FRAP) have been routinely used to demonstrate the total antioxidant potential of plant samples, and mechanism action of phyto-antioxidants^10^. However, sample preparation procedure for these assays is somewhat time-consuming and laborious^11^. Conversely, methyl viologen or paraquat-induced oxidative stress leaf disc assay is simple and does not require any sample preparation^12^. However, this assay provides limited information about physiological role, response and mechanism of action of phyto-antioxidants. In addition, paraquat is highly toxic and its use has been restricted in many countries, including the USA.

A number of leaf disc assays have been reported for growth measurements, to assess the accumulation of shikimate, and confirm the improved tolerance against oxidative stress in plants^13–15^. These leaf assays are mainly developed to understand the representative physiological response of plants to internal and external stimuli. Leaf disc assays have some advantages over whole plant assays, because they require less sample preparation time, rapid, and allow higher numbers of replicates^16^.

**Figure 1:**
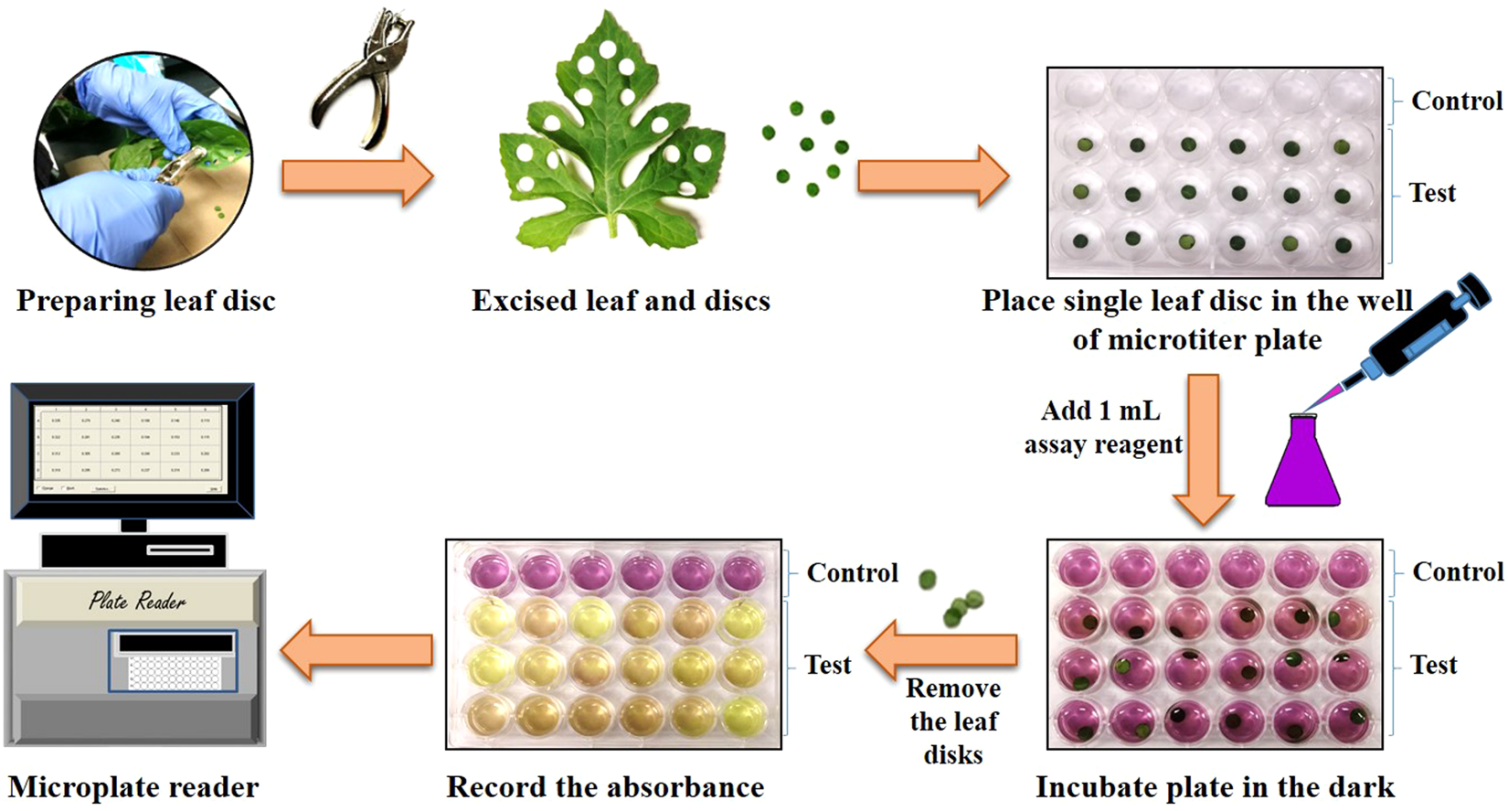
Workflow of rapid ABTS, DPPH, and PPR leaf disc assays for quick measurement of the antioxidant activity of plant samples.

In the present study, we have shown the free radical scavenging activity and reducing power of various excised leaf discs, and used this concept to develop DPPH, ABTS and PPR leaf disc assays. All three proposed leaf disc assays have a common workflow, schematically illustrated in Fig. 1. The fundamental principle behind each assay was that the antioxidants oozes out from the edges of the excised leaf disc into the reaction medium and scavanges free DPPH^•^ and ABTS^•+^ radicals and reduces KMnO_4_ to manganese dioxide (MnO_2_).

## Methods

### Chemicals

DPPH and KMnO_4_ were purchased from Sigma Aldrich (St. Louis, MO, USA). ABTS was obtained from Chem-Impex Int’l. Inc. (Bensenville, IL, USA). The magnesium sulfate heptahydrate (MgSO_4_. 7H_2_O) and zinc sulfate heptahydrate (ZnSO_4_. 7H_2_O) were procured from MCB Reagents and EM Science, an affiliate of Merck KGaA, Germany, respectively. All other chemicals used were of analytical grade.

### Instrumentation

The microplate reader (Synergy-HT, BioTek) and double-beam spectrophotometer (Hitachi, U-2900) was used to measure absorbance. Ultra-performance liquid chromatography coupled to an electrospray quadrupole time-of-flight mass spectrometer (UPLC/ESI-HR-QTOFMS) (Bruker Daltonics, Billerica, MA, USA) was used to identfy the phenolic compounds and measure the relative abundance in leaf disc suspended and cell-free extracts.

### Plant materials and preparation of leaf disc(s)

The fresh leafy vegetables such as spinach, kale, collards, and mustard were purchased from a local supermarket in College Station (TX, USA). Leaves of watermelon were obtained from greenhouse-grown plants. For bioassays, leaf discs of each plant material were prepared using a single hole punch with a diameter of 7 mm.

### Halopriming and growth chamber experiments

The diploid and triploid watermelon seeds (Origene Seeds Ltd., Rehovot, Israel) were primed for 14 h in 100 and 200 ppm solutions of magnesium sulfate (MgSO_4_) and zinc sulfate (ZnSO_4_). The unprimed and water soaked seeds were used as a control and hydroprimed treatment, respectively. Next day, the seeds were air dried, the unprimed, hydroprimed, and haloprimed seeds (10 seeds in triplicate under each treatment) were sown in a plug tray (200 square cells) filled with potting soil (Metro-mix 900, Sun Gro Horticulture, Seba Beach, Canada), and placed in a growth chamber at 70% relative humidity under a 14 h/10 h (light/dark, 28 °C/18 °C) photoperiod for germination and seedling development. After eight days, leaves of seedlings from each group were used for leaf disc assays.

### Optimization and validation of ABTS, DPPH, and PRP leaf disc assays

Initially, for all three assays, reagent concentration and reaction time of maximum reactivity was optimized using different leaf discs of spinach, kale, collards, mustard, and watermelon. The initial reagent concentrations were selected based on their absorbance close to 1.0 at respective wavelengths. For each assay, the final concentration of reagent and reaction time was optimized by considering parameters such as minimal reagent concentration, the shortest incubation time, and linearity of the reaction. Each assay was further validated for linearity and intra-assay precision. The calibration curves were established with 1–4 leaf discs as per the procedure described below. The plot of percent activity versus respective disc number(s)/weight of leaf disc(s) was used to calculate linear regression (r^2^). Similarly, intra-assay precision for each assay was calculated in three consecutive experiments and expressed as the percent coefficient of variation (%CV).

### ABTS leaf disc assay

The ABTS reagent was prepared by mixing equal amounts of aqueous 7.4 mM ABTS and 2.6 mM potassium persulfate solutions and allowed to react overnight in the dark^17^. The ABTS reagent was diluted to 1:40 with nano-pure water to obtain a working solution. A single leaf disc of size 7 mm was transferred to each well and 1 mL of the working ABTS solution was added. The plate was incubated for 30 min in the dark. Later on, leaf discs were removed from wells and absorbance was recorded in the microplate reader at 730 nm. The percent (%) ABTS radical scavenging activity was expressed as milligram (mg) of fresh weight (FW) of leaf disc using the following equation.

$$ \% \,{\rm{Radical}}\,{\rm{scavenging}}/{\rm{mg}}\,{\rm{FW}}\,{\rm{of}}\,{\rm{leaf}}\,{\rm{disc}}=(\frac{{A}_{Control}-{A}_{Sample}}{{A}_{Control}})\times (\frac{100}{FW\,of\,leaf\,disc\,(mg)})$$

Where, *A*_*Control*_ was the absorbance of the reagent control and *A*_*Sample*_ was the absorbance of the leaf disc suspended reagent solution after 30 min of incubation.

### DPPH leaf disc assay

The freshly prepared methanolic solution of DPPH reagent (0.102 mM) was used to assess the radical scavenging activity of leaf disc. An aliquot of 1 mL of DPPH solution was added into control and leaf disc-containing wells. After 10 minutes of incubation in the dark, leaf discs were removed from sample wells and the absorbance of the reagent solution was recorded in the microplate reader at 515 nm. The % DPPH radical scavenging activity per mg FW of leaf disc was calculated as mentioned above.

### PPR leaf disc assay

The fresh solution of 0.125 mM KMnO_4_ was used for this assay. For the assay, 1 mL of above KMnO_4_ reagent was added to each control and leaf disc containing sample well of 24-well microtiter plate and incubated for 30 min. Afterward, leaf discs were removed from sample wells, and the absorbance of the plate was measured at 525 nm in the microplate reader, and % PPR activity was calculated using above same % radical scavening activity formula.

### Antioxidant activity of cell-free extracts using conventional methods

The leaf samples of unprimed (control), hydroprimed and haloprimed watermelon seedling were crushed manually. A volume of 1 mL nano-pure water and methanol was added into 50 mg of crushed leaves to prepare aqueous and methanolic cell-free extracts, respectively. After vortexing (30 s), all tubes were sonicated for 60 min under chilled conditions, centrifuged at 10,621 × g for 10 min, and supernatants were collected for assays. The methanolic extract was used for DPPH assay, and the aqueous extract was used for ABTS and PPR assays. For *in vitro* DPPH, ABTS, and PPR antioxidant assays, 10 μL of the extract was diluted to 100 μL with nano-pure water or methanol in 96-well plate, and 100 μL of the respective reagent was added to each well^18^. The plates were kept in the dark for 5 min for ABTS and DPPH assays or kept in the dark for 10 min for PPR assays. The absorbance of the test sample and corresponding blank reaction mixtures was measured in the microplate reader for each assay at the respective wavelengths, and % activity was expressed in terms of mg FW of leaf^17,19,20^.

### UV-Vis spectroscopy and UPLC/ESI-HR-QTOFMS experiments

The methanolic and aqueous leaf disc suspended extracts of spinach, kale, collards, mustard, and watermelon were prepared by immersing a leaf disc of each plant material in 1 mL methanol and water for 10 min and 30 min, respectively. Leaf discs were removed, and extracts used for UV-Vis spectroscopy and UPLC/ESI-HR-QTOFMS analysis. UV-Vis scanning spectra of these extracts were recorded in the scanning range 200–800 nm. In addition, identification of some phenolic compounds and estimation of their relative abundances in various leaf discs and corresponding cell-free extracts were performed by UPLC/ESI-HR-QTOFMS. A volume of 2 μL was injected into the UPLC model 1290, Agilent, Santa Clara, CA, USA) and separation was achieved on rapid resolution Eclipse Plus C_18_ RRHD (1.8 μm, 50 × 2.1 mm) column. Phenolic compounds were separated using gradient mobile phases consists of (A). 0.1% aqueous formic acid, and (B). 0.1% formic acid in acetonitrile. Set gradient program for pump B was as follows: 0–2 min, 0%; 2–15 min, 0–80%; 15–18 min, 80–0%; 18–20 min, 0% B for 2 min. The column temperature was set at 35 °C. Mass spectra were acquired in positive mode using electrospray ionization (ESI) on a maXis impact mass spectrometer (Bruker Daltonics, Billerica, MA, USA). The mass spectrometer operating parameters were: nebulizer gas pressure, 2.8 bar; nebulizer gas flow, 8 L min^−1^; sheath nebulizer gas temperature, 220 °C; sheath gas heater temperature, 220 °C. DataAnalysis (version 4.3) software was used to process the data. UPLC chromatogram of each extract at 280 nm was used to generate comparative chromatographic fingerprint profiles. The identification of some phenolic compounds was achieved by matching maximum UV, absorptions, pseudomolecular ion mass values and MS/MS fragmentation patterns with data published in the literature^21–25^. UPLC/ESI-HR-QTOFMS peak area/mg FW of leaf material was used to calculate the relative abundance of some phenolic compounds in each extract.

### Statistical analysis

Statistical evaluation of data was performed using Microsoft Excel (2010) and SPSS version 13 (SPSS, Inc.). One-way analysis of variance (ANOVA) with Tukey’s post-hoc test was performed to determine inter-group differences.

## Results

### Optimization of reagent concentration and reaction time for maximum reactivity

**Figure 2:**
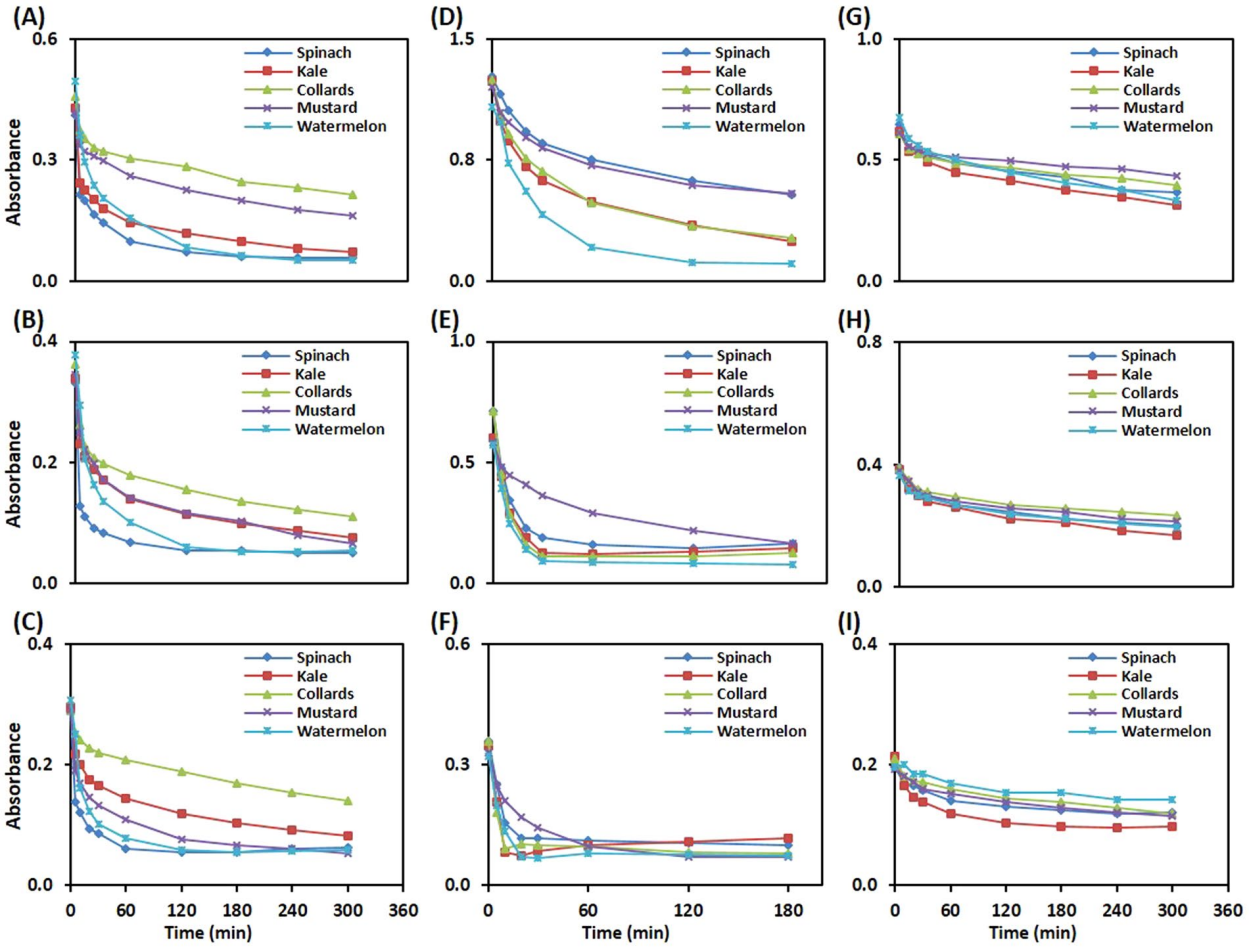
Optimization of the reagent concentration and time of maximum reactivity for aqeous and methanolic ABTS, DPPH, and PPR leaf disc assays. (**A**–**C**) Shows the time-dependent decay of ABTS^•+^ radical by various leaf discs at different reagent dilution ratios, 1:30 (**A**), 1:40 (**B**) and 1:50 (**C**) with water at wavelength 725 nm. (**D**–**F**) Indicates time-dependent scavenging of DPPH radical by different leaf discs in a methanolic DPPH solution of concentrations, 0.204 (**D**), 0.102 (**E**) and 0.051 mM (**F**) at wavelength 515 nm. The various leaf discs-based reduction of a KMnO_4_ solution of different concentrations, 0.500 (**G**), 0.250 (**H**) and 0.125 mM (**I**) at wavelength 525 nm. The value at each time point represents the mean absorbance of technical replicates (n = 3–6).

The leaf discs from spinach, kale, collards, mustard, and watermelon were used to optimize the reagent concentration and reaction time for each assay. For the ABTS leaf disc assay, the reagent concentration was initiated with 1:30 diluted solution, based on its optimal absorbance less than 1.0 at wavelength 730 nm (Fig. S1). All leaf discs showed time-dependent decay in the absorbance at various dilutions (1:30, 1:40 and 1:50) of ABTS reagent with nano-pure water (Fig. 2A–C). The observed ABTS^•+^ radical scavenging potential of leaf discs was linear at the beginning and found to attend plateau in a later phase of incubation, up to 5 h. Among studied vegetables, spinach leaf discs showed maximum decay of ABTS^•+^ radical upto incubation of 120 min at dilution ratios 1:40 (B) and 1:50 (C) of ABTS reagent. To give a broader window of detection, the dilution ratio of 1:40 of ABTS reagent was confirmed as an optimal concentration for leaf disc assays for the assessment of linearity.

To optimize reagent concentration and incubation time for the DPPH leaf disc assay, DPPH reagent was prepared in methanol at three concentrations, 0.051, 0.102, and 0.204 mM, based on literature reports^17,26,27^. The results of time-dependent decay of absorbance of DPPH solutions (0.051, 0.102, and 0.204 mM) by various leaf discs at 515 nm over a period of 3 h are depicted in Fig. 2(D–F). At 0.051 mM DPPH reagent concentration (Fig. 2F), the majority free radicals were scavenged within 20 min of incubation. For 0.102 mM DPPH reagent concentration (Fig. 2E), the observed time for maximum scavenging of DPPH radical by leaf discs was 30 min. To achieve a sufficient limit of detection, 0.102 mM DPPH and corresponding time of maximum reactivity (30 min) were further used for assessment of linearity. At this concentration, the observed absorbance of DPPH solution was also close to 1 at wavelength 515 nm (Fig. S2).

To optimize reagent concentration and incubation time for the PPR leaf disc assay, tests were carried out at 0.500, 0.250, and 0.125 mM KMnO_4_ solutions at different time intervals over a period of 300 min. The initial concentration of the KMnO_4_ solution was fixed based on the absorbance of the solution close to 1 at 525 nm (Fig. S3). All leaf discs showed a time-dependent reduction of the KMnO_4_ reagent at the tested concentrations (Fig. 2G–I). At 0.500 and 0.250 mM, all leaf discs showed a constant decrease in the absorbance at 515 nm over a period of 5 h. However, at 0.125 mM, leaf discs of kale showed a maximum reduction of KMnO_4_ up to 2 h of incubation. Based on these findings, 0.125 mM KMnO_4_ was confirmed as an optimal reagent concentration for PPR leaf disc assay, and linearity was assessed with the corresponding time of 2 h incubation.

### Linearity and intra-assay precision

For each assay, the four-points (1–4 leaf disc(s)) calibration was conducted using leaves of spinach, kale, collards, mustard, and watermelon at optimized reagent concentrations and in a range around the corresponding incubation times. All three assays showed leaf disc-dependent colour change (Fig. S4). The linearity of the DPPH leaf disc assay was assessed at three incubation times, 10, 20, and 30 min. For the ABTS and PPR leaf disc assays, calibration curves were obtained at 30, 60, and 120 min of incubation. In the DPPH assay, good linearity (r^2^ = 0.767–0.941) was observed for the majority of leaf discs at 10 min of incubation (Table 1). The ABTS (r^2^ = 0.679–0.989) and PPR (r^2^ = 0.660–0.941) leaf disc assays had good linearity at 30 min of incubation. The observed linearity of all three leaf disc assays were found to be dependent on incubation time, and decreased with increasing incubation time (Tables S1 and S2).

**Table 1:** Linearity and accuracy studies of ABTS*, DPPH** and PPR* leaf disc assays.

Leaf material	Leaf disc range	Correlation coefficient (r^2^)			Intra-assay precision (%CV)		
		ABTS	DPPH	PPR	ABTS	DPPH	PPR
Spinach	1–4	0.857	0.913	0.660	41.4	7.73	7.55
Kale	1–4	0.954	0.767	0.887	12.4	11.1	26.2
Collards	1–4	0.983	0.941	0.935	8.29	29.5	23.6
Mustard	1–4	0.679	0.849	0.941	4.40	27.2	12.5
Watermelon	1–4	0.989	0.881	0.871	6.92	5.13	4.56

*Results at 30 min of incubation time.

**Results at 10 min of incubation time.

Results of intra-assay precision (%CV) tests of the DPPH (at 10 min incubation), ABTS, and PPR (at 30 min incubation) leaf disc assays are summarized in Table 1. All studied leaf materials had %CVs value within the acceptable limits. However, in some cases, the observed %CV value was higher than 15% due to incubation time influenced %CV value (Tables S1 and S2). In addition, the correlation between the weight of the disc(s) with % activity was studied to understand the role of variation in weight of discs on observed % activity in each assay. The results of correlation study between the weight of various leaf disc(s) and number of the disc(s) with % activity in ABTS, DPPH, and PPR leaf disc assays are shown in Table 1 and Fig S5. In many cases, the weight of leaf disc(s) had a stronger correlation with % activity compared to the correlation between numbers of leaf disc(s) with % activity.

### UV-Vis spectroscopy and UPLC/ESI‐HR‐QTOFMS experiments

**Figure 3:**
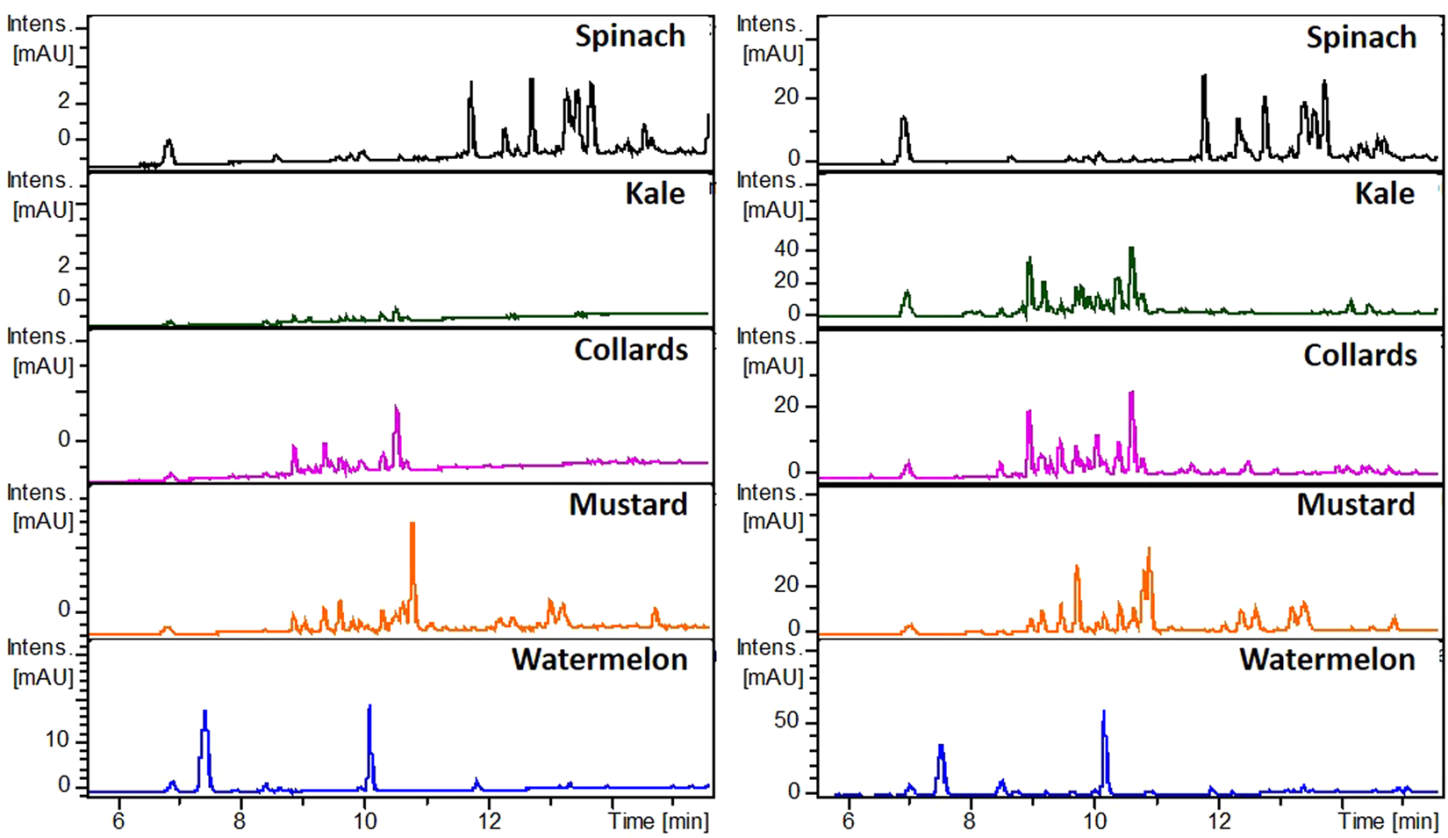
Comparative UPLC chromatographic profiles of leaf disc suspended and cell-free methanolic extracts of various plant materials at 280 nm. The difference in absorption intensities of leaf disc suspended and cell-free extracts are not only linked with the different amount of leaf tissue used for extraction, but also with diverse extraction efficacy in both the methods.

These studies were undertaken to understand the mechanisms of radical scavenging and reducing power, as well as phyto-antioxidants extraction efficacies of leaf discs and cell-free extracts methods in water and methanol. Scanning absorption spectra of various leaf discs suspended in methanol extracts showed that lipophilic and hydrophilic compounds, including chlorophyll, diffuse quickly from the leaf disc in the methanolic medium (Fig. S6). By contrast, relatively small amounts of phyto-antioxidants, mainly hydrophilic compounds, come out from the leaf discs in the aqueous medium after 30 min of incubation (Fig. S6). Further, leaf discs suspended and respective cell-free extracts in water and methanol were analysed by UPLC/ESI‐HR‐QTOFMS. The comparative chromatographic profiles at 280 nm are shown in Figs 3 and S7. Interestingly, leaf disc suspended methanolic extract had almost the same chromatographic fingerprints with their corresponding cell-free extracts (Fig. 3). However, suspended leaf disc aqueous extracts had distinct UPLC chromatographic profiles at 280 nm compared with those of cell-free extracts (Fig. S7).

**Figure 4:**
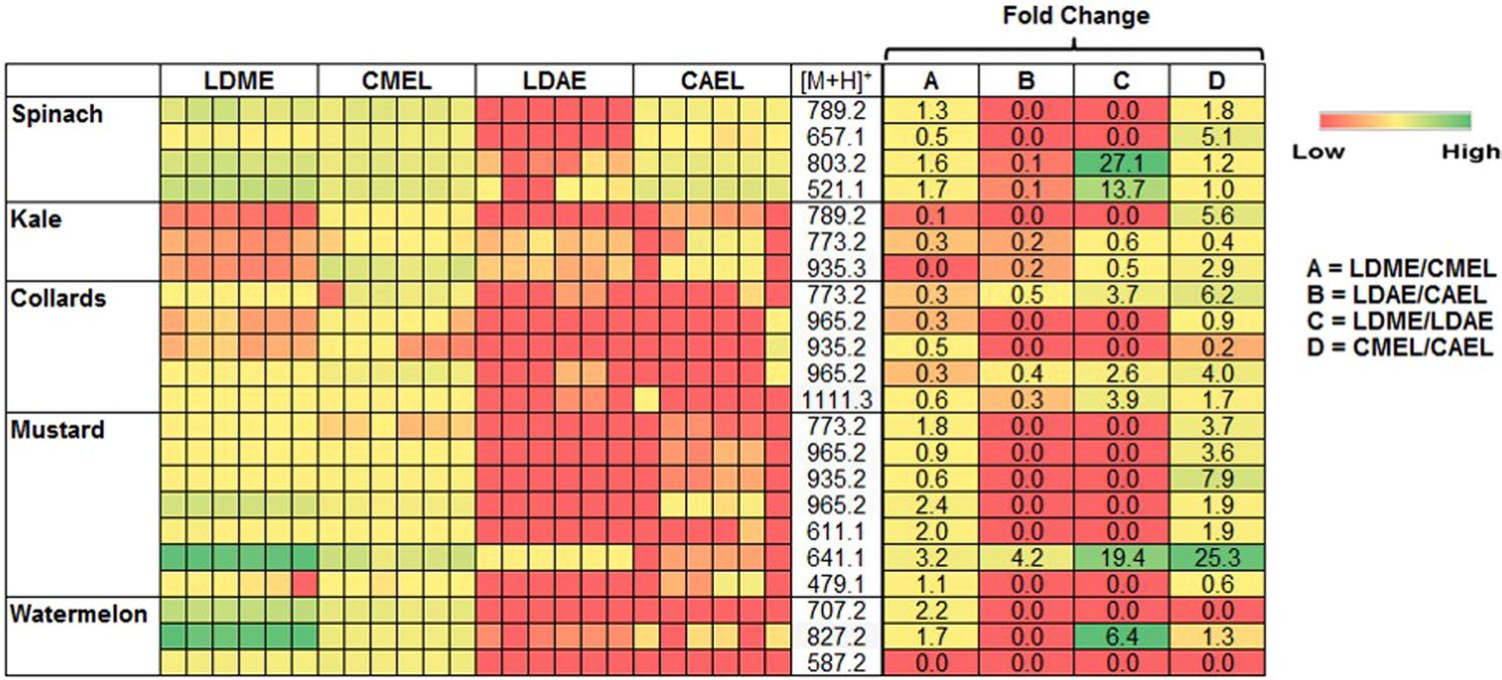
UHPLC/ESI‐HR‐QTOFMS based relative abundances of selected phenolic compounds present in various extracts of different leaf materials (CMEL – cell-free methanolic extract of leaf, LDME - leaf disc suspended methanolic extract, CAEL- cell-free aqueous extract of leaf, LDAE- leaf disc suspended aqueous extract). Data represent the average of three biological replicates.

The UPLC/ESI‐HR‐QTOFMS data were further used to identify some phenolic compounds and estimate their abundance in various suspended leaf disc and cell-free extracts. The identified phenolic compounds from leaves of spinach, kale, collards, mustard, and watermelon are tabulated in Table 2. Furthermore, the relative abundance and extraction fold change of each phenolic compound in various suspended leaf disc and cell-free extracts are shown in Fig. 4. The methanolic extracts had higher abundance of phenolic compounds than that of aqueous extracts. However, the extraction efficacy of some selected phenolic compounds in the same solvent was not constant in the suspended leaf disc and cell-free extracts. This might be due to the physical traits of the leaf, such as the surface composition, shape, margin, vein structure and among others. Interestingly, the abundance of some phenolic compounds was found to be higher in leaf disc suspended extracts than in cell-free extracts (fold change >1).

**Table 2:** Identification of phenolic compounds from various leaf materials by UHPLC/ESI‐HR‐QTOFMS.

Leaf material	Rt (min)	UV λ_max_ (nm)	Experi-mental mass (*m/z*)	MS/MS fragments	Compound	Molecular formula	Theoretical mass (*m/z*)	Mass error (ppm)	ref.
Spinach	11.8	255, 350	789.2270	333	Patuletin-3-O-β-D-glucopyranosyl-(1 → 6)-[β-D-apiofuranosyl-(1 → 2)]-β-D-glucopyranoside	C_33_H_40_O_22_	789.2083	−9.5	^25^
	12.3	255, 350	657.1630	347, 333	Patuletin-3-O-β-D-glucopyranosyl-(1 → 6)-β-Dglucopyranoside	C_28_H_32_O_18_	657.1661	−4.7	^25^
	12.8	255, 350	803.2190	421, 347	Spinacetin-3-O-β-D-glucopyranosyl-(1 → 6)-[β-D-apiofuranosyl-(1 → 2)]-β-D-glucopyranoside	C_34_H_42_O_22_	803.2240	−6.3	^25^
	18.0	254, 278, 340	521.0931	345, 330	5, 3, 4 -Trihydroxy-3-methoxy-6:7-methylendioxyflavone-4 - β-D-glucuronide	C_23_H_20_O_14_	521.0926	1.0	^25^
Kale	8.4	254, 340	789.2081	425, 347, 303, 204, 132, 84	Quercetin-3-diglucoside-7-glucoside	C_33_H_40_O_22_	789.2084	−0.4	^22, 23^
	8.9	258, 338	773.2136	347, 287, 186, 132, 84	Kaempferol-3-diglucoside-7-glucoside	C_33_H_40_O_21_	773.2135	0.1	^22, 23^
	9.2	258, 338	934.2633	633, 571, 449, 347, 287, 84	Kaempferol-3-diglucoside-7-diglucoside	C_39_H_50_O_26_	934.2585	5.2	^22, 23^
Collards	8.9	268, 344	773.2206	541, 347, 287	Kaempferol-3-diglucoside-7-glucoside	C_33_H_40_O_21_	773.2135	9.2	^21, 22^
	9.5	253, 268, 338	965.2561	449, 355, 287, 193, 133, 84	Kaempferol-3-hydroxyferuloyldiglucoside-7-glucoside	C_43_H_48_O_25_	965.2557	0.4	^21, 22^
	9.7	338	935.2561	409, 287, 163, 84	Kaempferol-3-caffeoyldiglucoside-7-glucoside	C_42_H_46_O_24_	935.2451	11.7	^21, 22^
	10.1	338	965.2590	409, 303	Quercetin-3-O-feruloyldiglucoside-7-glucoside	C_43_H_48_O_25_	965.2557	3.4	^21, 22^
	10.5	254, 268, 334	1111.3239	949, 532, 449, 287, 177, 84	Kaempferol-3-feruloyltriglucoside-7-glucoside	C_49_H_58_O_29_	1111.313	9.2	^21, 22^
Mustard	8.9	268, 344	773.2206	541, 347, 287	Kaempferol-3-diglucoside-7-glucoside	C_33_H_40_O_21_	773.2135	9.2	^21, 22^
	9.5	253, 268, 338	965.2625	449, 355, 287, 193, 161, 133, 84	Kaempferol-3-hydroxyferuloyldiglucoside-7-diglucoside	C_43_H_48_O_25_	965.2557	7.0	^21, 22^
	9.7	253, 268, 338	935.2463	409, 287, 163, 135, 84	Kaempferol-3-caffeoyldiglucoside-O-glucoside	C_42_H_46_O_24_	935.2451	1.2	^21, 22^
	10.1	253, 338	965.2590	409, 303	Quercetin-3-O-feruloyldiglucoside-7-O-glucoside	C_43_H_48_O_25_	965.2557	3.4	^21, 22^
	10.5	253, 268, 334	611.1577	287	Kaempferol-3-diglucoside	C_27_H_30_O_16_	611.1606	−4.8	^21, 22^
	10.8	268, 338	641.1695	317	Isorhamnetin-3-diglucoside	C_28_H_32_O_17_	641.1712	−2.7	^21, 22^
	14.8	254, 266, 352	479.1155	317	Isorhamnetin-3-glucoside	C_22_H_22_O_12_	479.1184	−6.1	^21, 22^
Watermelon	7.5	246, 330	707.2320	365, 163, 89	Unknown	—	—	—	—
	8.5	n.d.	827.3534	425, 319, 157, 107	Unknown	—	—	—	—
	15.7	250, 332	587.2183	319, 163, 107, 89	Unknown	—	—	—	—

n.d.- indicates not detecte.

### Comparative antioxidant potentials in leaf disc and conventional assays

**Figure 5:**
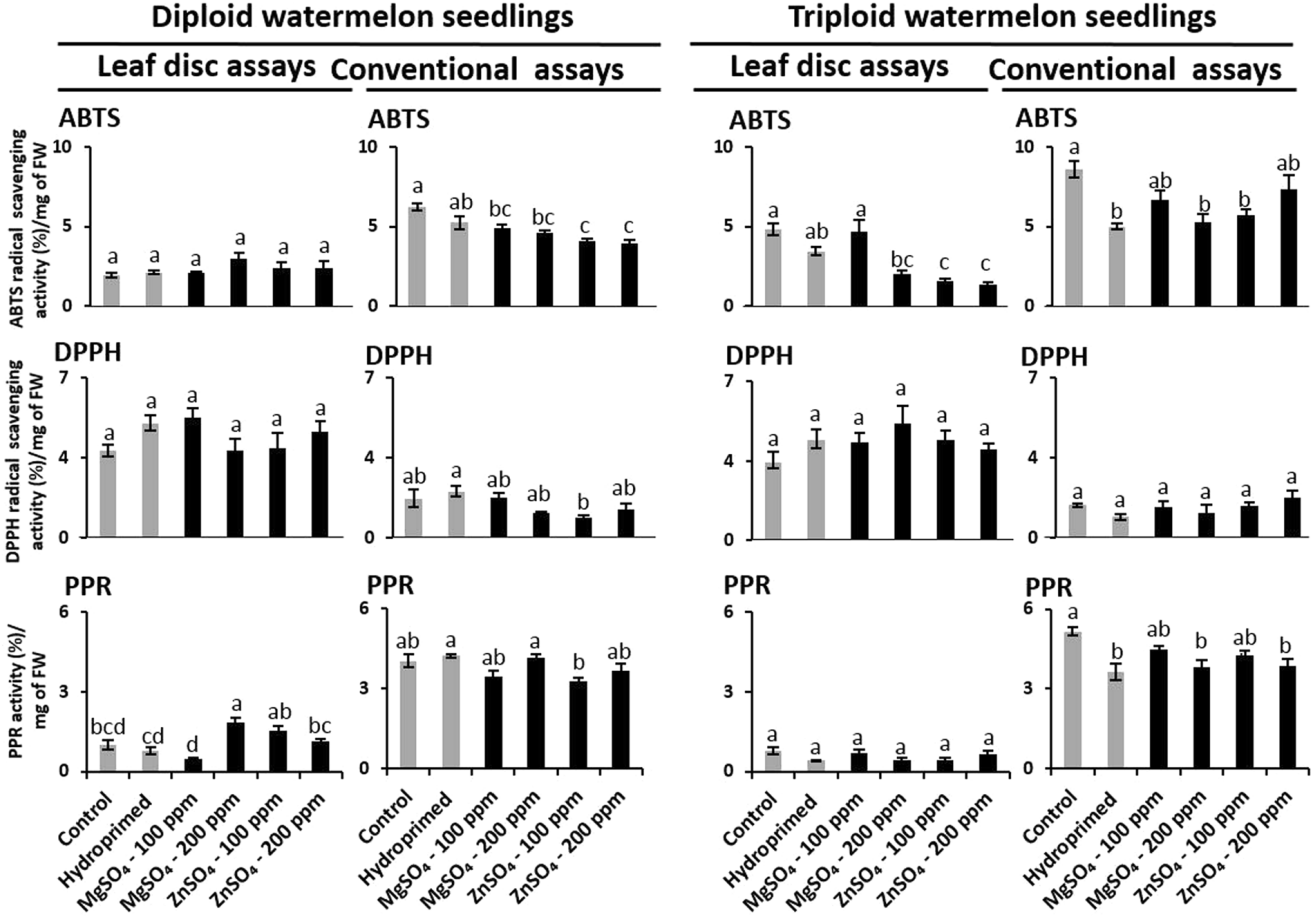
Comparative antioxidant activity of leaf samples of unprimed, hydroprimed, and haloprimed (100 and 200 ppm of magnesium sulfate (MgSO_4_) and zinc sulfate (ZnSO_4_)) diploid and triploid watermelon seedlings in the leaf disc and conventional assays. Values are expressed as means ± SE of three independent biological replicates and two three technical replications. Different superscript letters in the row indicate significant differences according to Tukey’s test (*P* < 0.05).

The suspended leaf disc and cell-free extracts of seedling leaves of unprimed, hydroprimed, and haloprimed (100 ppm and 200 ppm solutions of MgSO_4_ and ZnSO_4_) diploid and triploid watermelon varieties were used to compare the antioxidant levels in leaf disc and conventional assays. Figure 5 shows results of the comparative antioxidant activities in ABTS, DPPH, PPR leaf disc and conventional assays of these samples. We found that haloprimed treatments significantly altered the antioxidant levels of diploid and triploid watermelon seedlings in leaf disc and conventional assays. However, the results of leaf disc and conventional assays were not comparable. Interestingly, both varieties showed higher DPPH radical scavenging activity in leaf disc assays than in the conventional assay. However, compare to conventional assays, low antioxidant values were found in ABTS and PPR leaf disc assays in both varieties.

## Discussion

Based on chemical and biological mechanisms, several antioxidant activity assays have been reported to measure antioxidant potential of plant samples to obtain qualitatively and semi-quantitativelydata^3,18,27–29^. However, each of these assays have limitations in terms of accuracy, quantification, and applicability; therefore, multiple assays are usually recommended to measure the antioxidant potential of plant samples^3,10^. In view of this, three leaf disc assays were developed for a quick measurement of antioxidant potential of plant samples. Similar to conventional assays, measurement of the total antioxidant potential of plant samples was also practically impossible in the proposed leaf disc assays, because of the intactness of the leaf tissue, surface morphology, and extraction efficacy of solvents. Therefore, the developed leaf disc assays can be used for qualitative and comparative antioxidant assessment.

Based on the mechanism, antioxidant activity assessment methods are broadly classified into two types: single electron transfer (SET) and hydrogen atom transfer (HAT)-based assays^30^. However, certain assays such as DPPH and ABTS involve both SET and HAT mechanisms. DPPH is usually soluble in organic solvents (alcohols) whereas, ABTS is soluble in aqueous and organic solvents. Because of this, the DPPH assay represents the antioxidant potential of more lipophilic compounds, whereas, ABTS assay represents hydrophilic and lipophilic compounds^31^. In general, the ABTS assay better estimates the antioxidant capacity of foods, particularly fruits, vegetables, and beverages compared to the DPPH method^32,33^. However, we found that the DPPH leaf disc assay is more rapid and estimates more antioxidant activity than the ABTS assay. This may be due to alcoholic medium of the DPPH reagent, in which both hydrophilic and lipophilic antioxidants including chlorophyll, diffuse from the edges of the excised leaf disc and react with the DPPH^•^ radical. Conversely, when the ABTS^•+^ radical is in the aqueous medium, it is mainly inactivated by water-soluble antioxidants that diffused from the edges of the excised leaf disc into the aqueous medium. Our scanning absorption spectra of methanolic and water extracts substantiated these facts (Fig. S6). Furthermore, results of UPLC/ESI‐HR‐QTOFMS based profiling and relative abundance of certain phenolics showed that methanol is the best medium to extract the comparable or even more antioxidants than that of cell-free extract. This could be the reason that we found high radical scavenging activity in the DPPH leaf disc assay compared with the conventional assay. Conversely, dilution of ABTS reagent with methanol was also found to remarkably increase the radical scavenging activity of the ABTS leaf disc assay, even higher than DPPH leaf disc assay (Fig. S8). However, the proposed ABTS leaf disc assay is designed to study the radical scavenging activity of hydrophilic antioxidants of leafy materials.

Among SET-based assays, FRAP (ferric reducing antioxidant power) and copper reduction assay (CUPRAC) are commonly used to measure the reducing power of plant samples^29^. However, each of these methods has advantages and disadvantages^34^. Most notably, these assays contain a multicomponent reagent and need to perform at specific pH. Therefore, we developed a simple PPR assay to understand the reducing potential of leaf discs. KMnO_4_ is an ecofriendly compound as well as known for its strong oxidizing property. In addition, the aqueous KMnO_4_ solution has considerable stability over a pH range of 5.8–8.0 (Fig. S9). Cacig and co-workers^19^ initially used a KMnO_4_ reduction method to report the antioxidant potential of plant extracts. However, this method has not been fully optimized in terms of reagent concentration and reaction time. For the first time, we have optimized the KMnO_4_ reagent concentration and optimal reaction time. In the PPR leaf assay, leaf disc antioxidants reduce permanganate ion (+7) into manganese dioxide (+4) by transferring three electrons at neutral pH (Fig. S10). Under the regular conditions, manganese dioxide is water-insoluble, usually appearing as a brown precipitate in permanganate reduction reactions. However, in the proposed PPR leaf disc assay, the pink KMnO_4_ solution was reduced into a yellow-brown transparent solution. As reported earlier, this sort of end product of reaction could be due to the formation of other forms (e.g. nano-form) of colloidal manganese dioxide, which do not form a precipitate over the period of incubation^35^. However, we found that the formation of this form of manganese dioxide at the end of the PPR leaf disc assay was mainly dependent on the initial concentration of the KMnO_4_ solution and the number of leaf discs used for the assay.

In the present study, time and concentration of reagent for each leaf assay were optimized using leafy vegetables, as they are the excellent source of a wide range of natural antioxidants such as vitamin C, carotenoids, and phenolics. The antioxidant potential of vitamin C, carotenoids, and phenolics is mainly due to their electron-rich structures in the form of oxidizable double bonds and hydroxyl groups^36^. For each leaf disc assay, time of incubation and concentration of reagent was defined by considering parameters like minimal concentration of reagent, the shortest incubation time, and linearity of reaction. In these assays, observed radical scavenging and reducing power ability for each leaf disc was initially linear with incubation time, and found to attend plateau at the maximum time of reactivity. In addition, the linearity of each of these assays was found to be greatly influenced by incubation time, which considerably reduced at the time of maximum reactivity due to the attainment of the reaction plateau. Several authors have demonstrated that it is important to measure reaction time at the steady state instead of at a fixed time, to avoid underestimation of results^34,37^. Therefore, based on maximum linearity, we fixed optimal incubation time for each assay. Herein, we also found that the weight of leaf disc has a considerably strong correlation with % activity, some places more than that of the number of leaf discs. This observation underlines the importance of consideration of weight while expressing the final results of the antioxidant assay. In addition, there is also need to express results at the equal platform in comparative antioxidant activity evaluation studies. Therefore, results of proposed leaf disc assays were expressed in term of percent activity per mg of F.W. of leaf disc. As we know that morphology and uniformity of the same leaf are not equal. Therefore each of these assays, maximum number of technical replicates need to be considered in order to obtain more accurate data.

The results of halopriming studies were promising and confirmed that DPPH leaf disc assay can be alternatively used for qualitative and comparative assessment of the antioxidant potential of leaf material. On the other hand, the observed antioxidants levels in ABTS and PPR leaf disc assay were lower than that of conventional methods. However, these assays could have considerable applications in the comparative antioxidant activity measurement studies.

## Conclusion

Herein, For the first time, we have developed novel DPPH, ABTS, and PPR leaf disc assays for comparative assessment of antioxidant potential of plant samples. The DPPH leaf disc assay estimated higher radical scavenging activity than that of conventional methods. ABTS and PPR leaf disc assays measured relatively lower activity than that of conventional methods. All together, the proposed assays are simple and rapid, and can be selectively and alternatively used to measure the qualitative and comparative antioxidant potential of leaf samples (mainly of intraspecies) from food and plant science investigations.

## Supplementary information

Supplementary Data
