# Supplementary material for: Leaf Disc Assays for Rapid Measurement of Antioxidant Activity
Source: Sci Rep. 2019 Feb 13;9:1884. doi: 10.1038/s41598-018-38036-x (PMC6374478; doi:10.1038/s41598-018-38036-x)
Supplement: Supplementary file 1 — Supplementary Data [file 41598_2018_38036_MOESM1_ESM.pptx]

## Slide 1
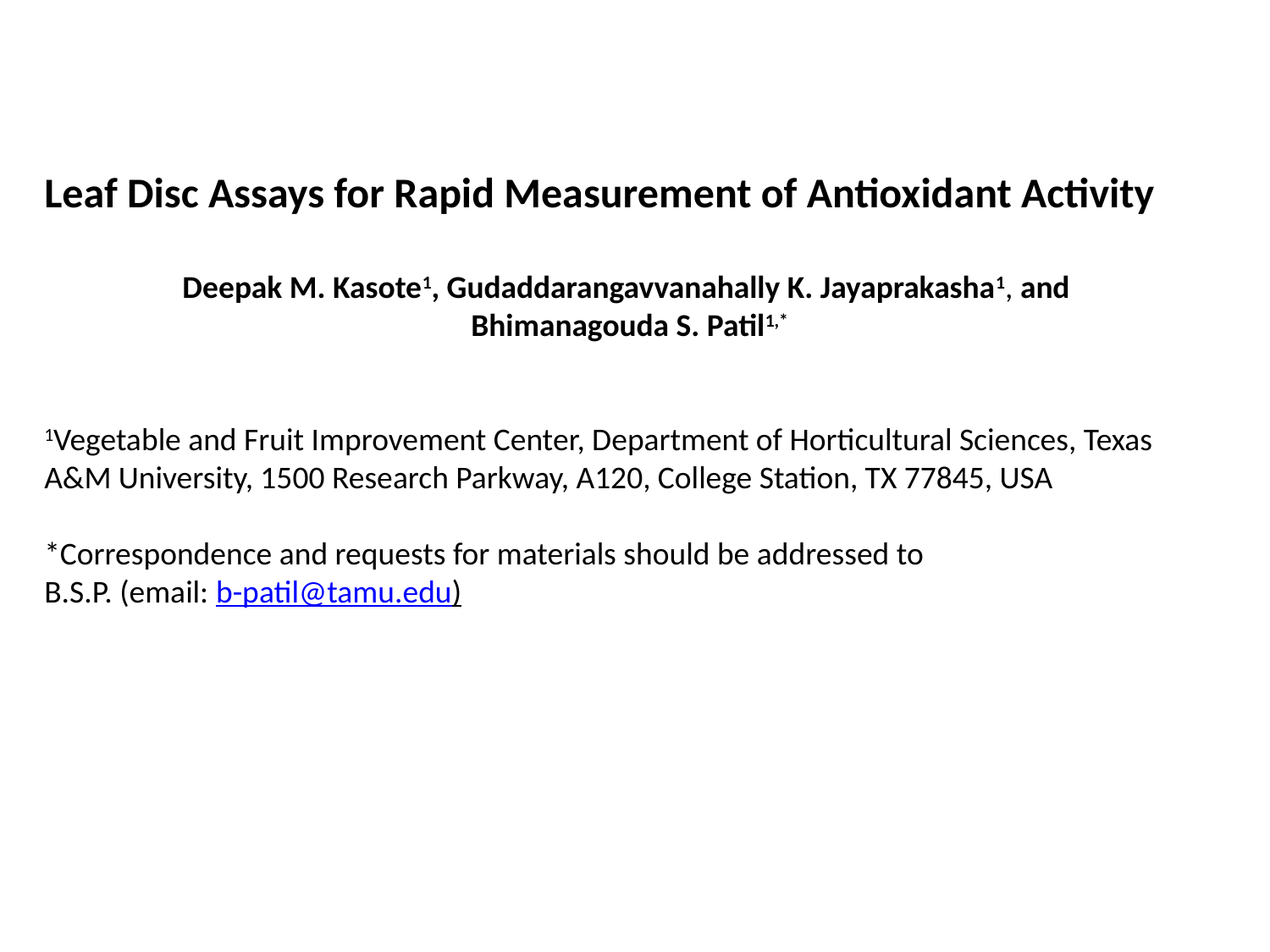

Leaf Disc Assays for Rapid Measurement of Antioxidant Activity
Deepak M. Kasote1, Gudaddarangavvanahally K. Jayaprakasha1, and
Bhimanagouda S. Patil1,*
1Vegetable and Fruit Improvement Center, Department of Horticultural Sciences, Texas A&M University, 1500 Research Parkway, A120, College Station, TX 77845, USA
*Correspondence and requests for materials should be addressed to
B.S.P. (email: b-patil@tamu.edu)

## Slide 2
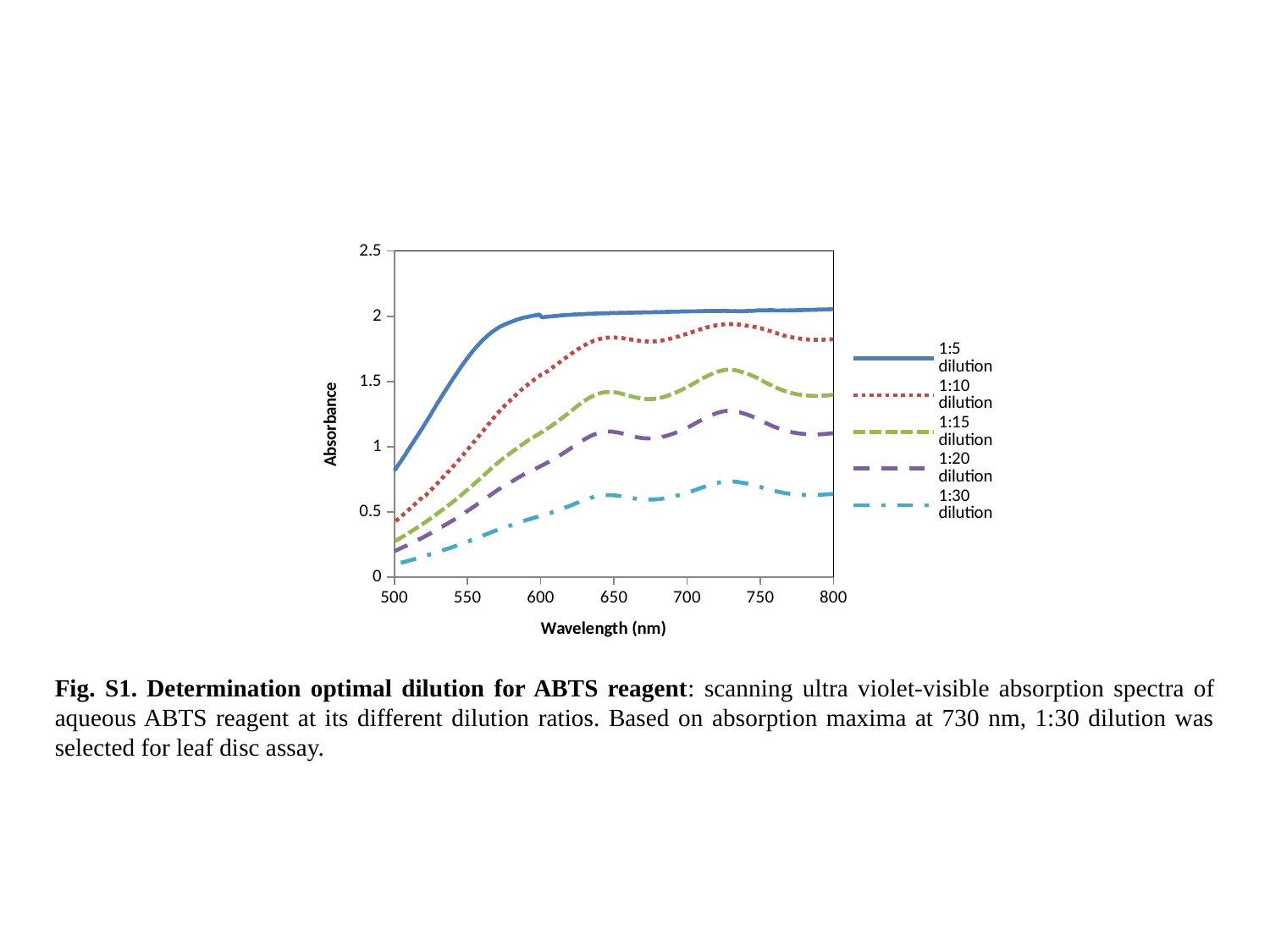

### Chart
| Category | 1:5 dilution | 1:10 dilution | 1:15 dilution | 1:20 dilution | 1:30 dilution |
|---|---|---|---|---|---|Fig. S1. Determination optimal dilution for ABTS reagent: scanning ultra violet-visible absorption spectra of aqueous ABTS reagent at its different dilution ratios. Based on absorption maxima at 730 nm, 1:30 dilution was selected for leaf disc assay.

## Slide 3
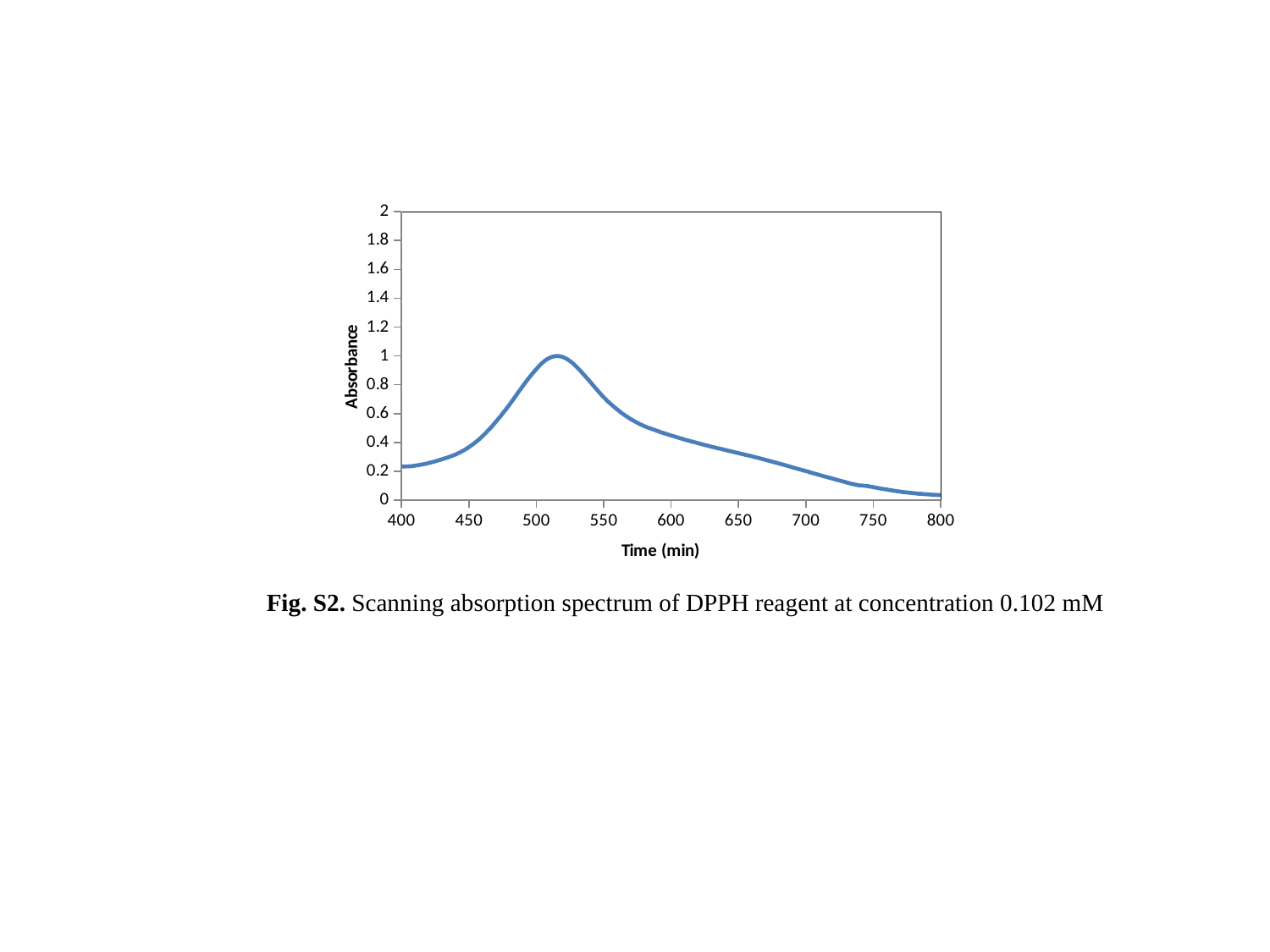

### Chart
| Category | 40 mg L⁻¹ |
|---|---|Fig. S2. Scanning absorption spectrum of DPPH reagent at concentration 0.102 mM

## Slide 4
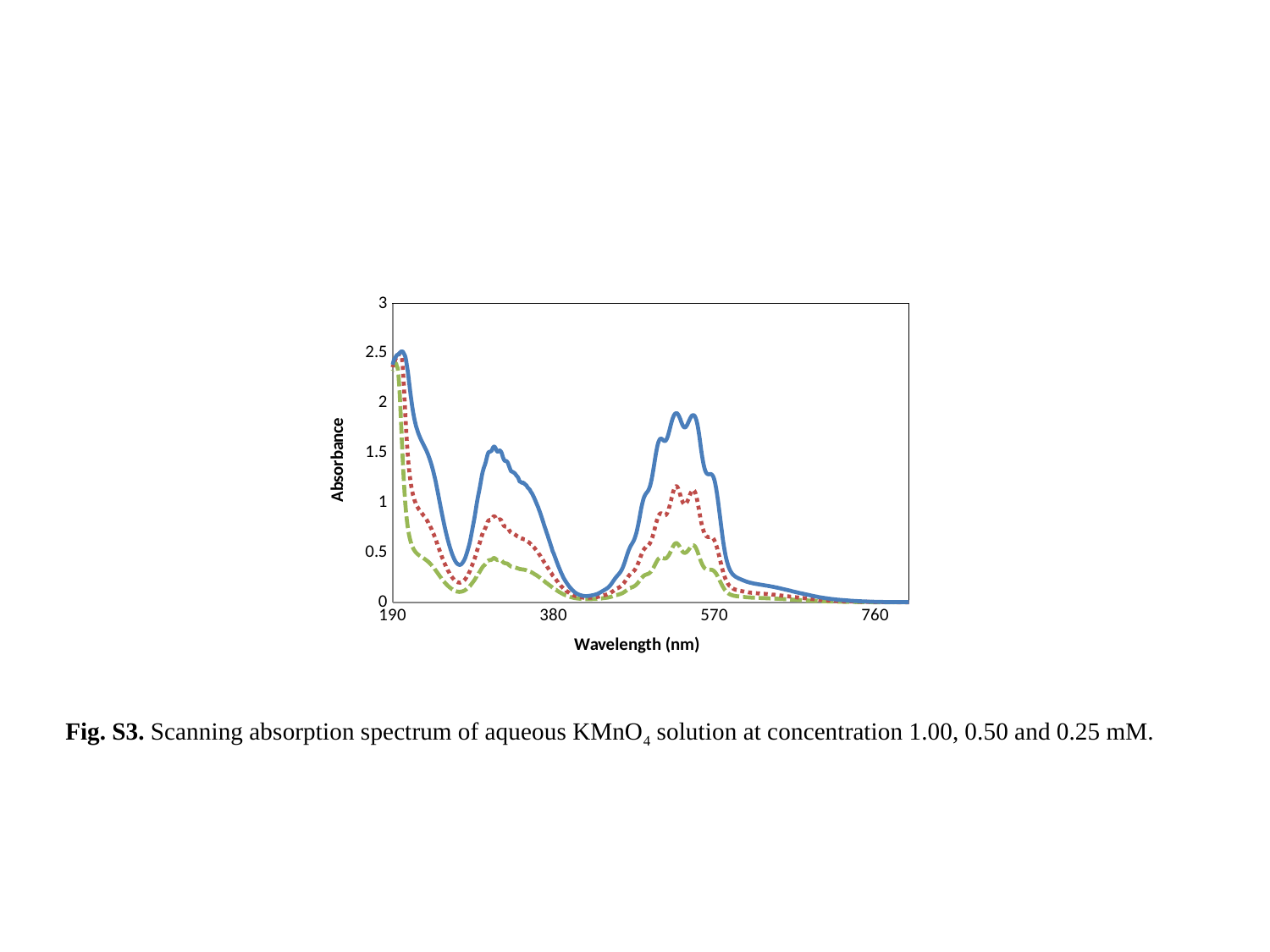

### Chart
| Category | 1 mM | 0.5 mM | 0.25 mM |
|---|---|---|---|Fig. S3. Scanning absorption spectrum of aqueous KMnO4 solution at concentration 1.00, 0.50 and 0.25 mM.

## Slide 5
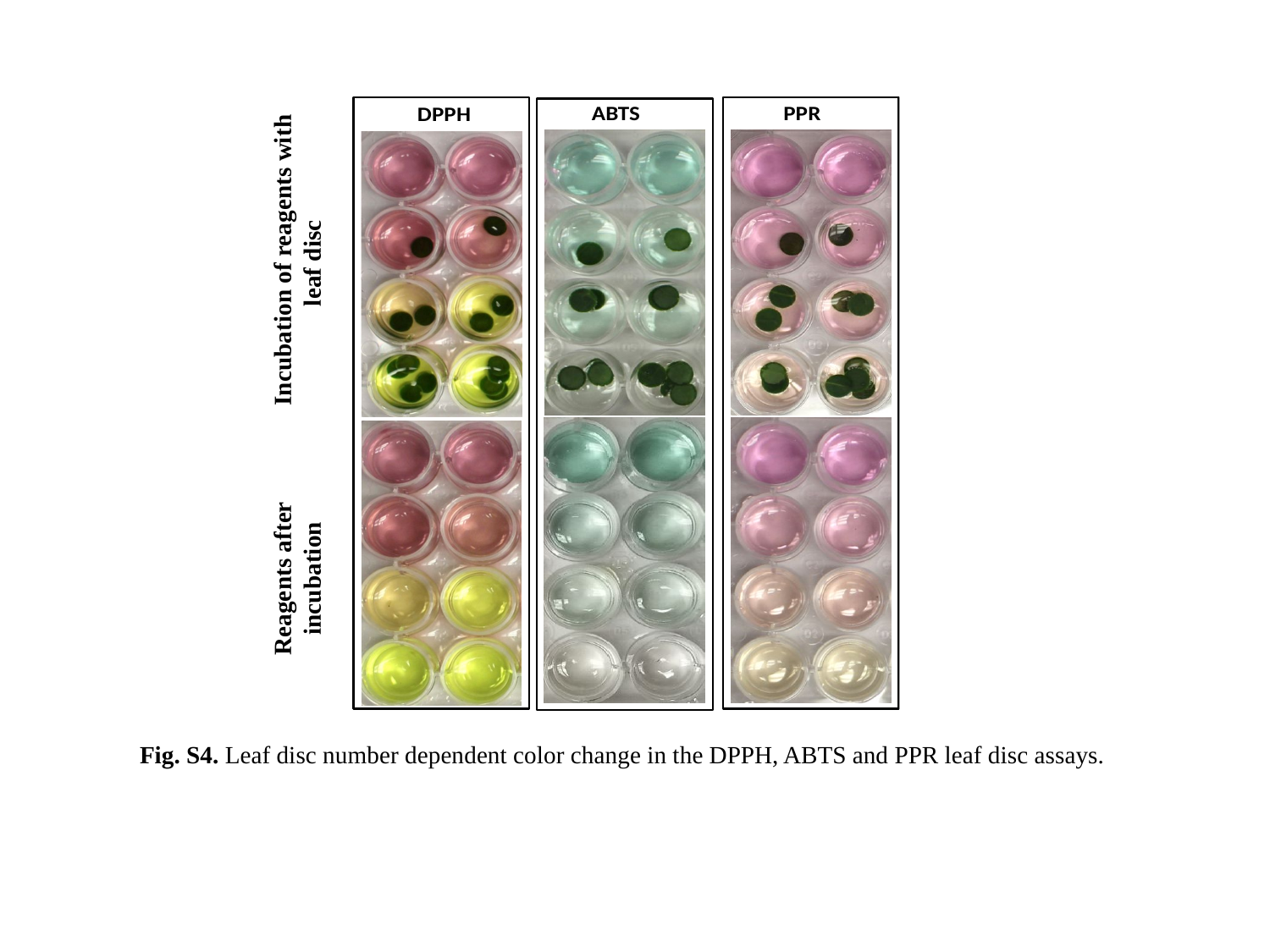

Incubation of reagents with leaf disc
Reagents after incubation
Fig. S4. Leaf disc number dependent color change in the DPPH, ABTS and PPR leaf disc assays.

## Slide 6
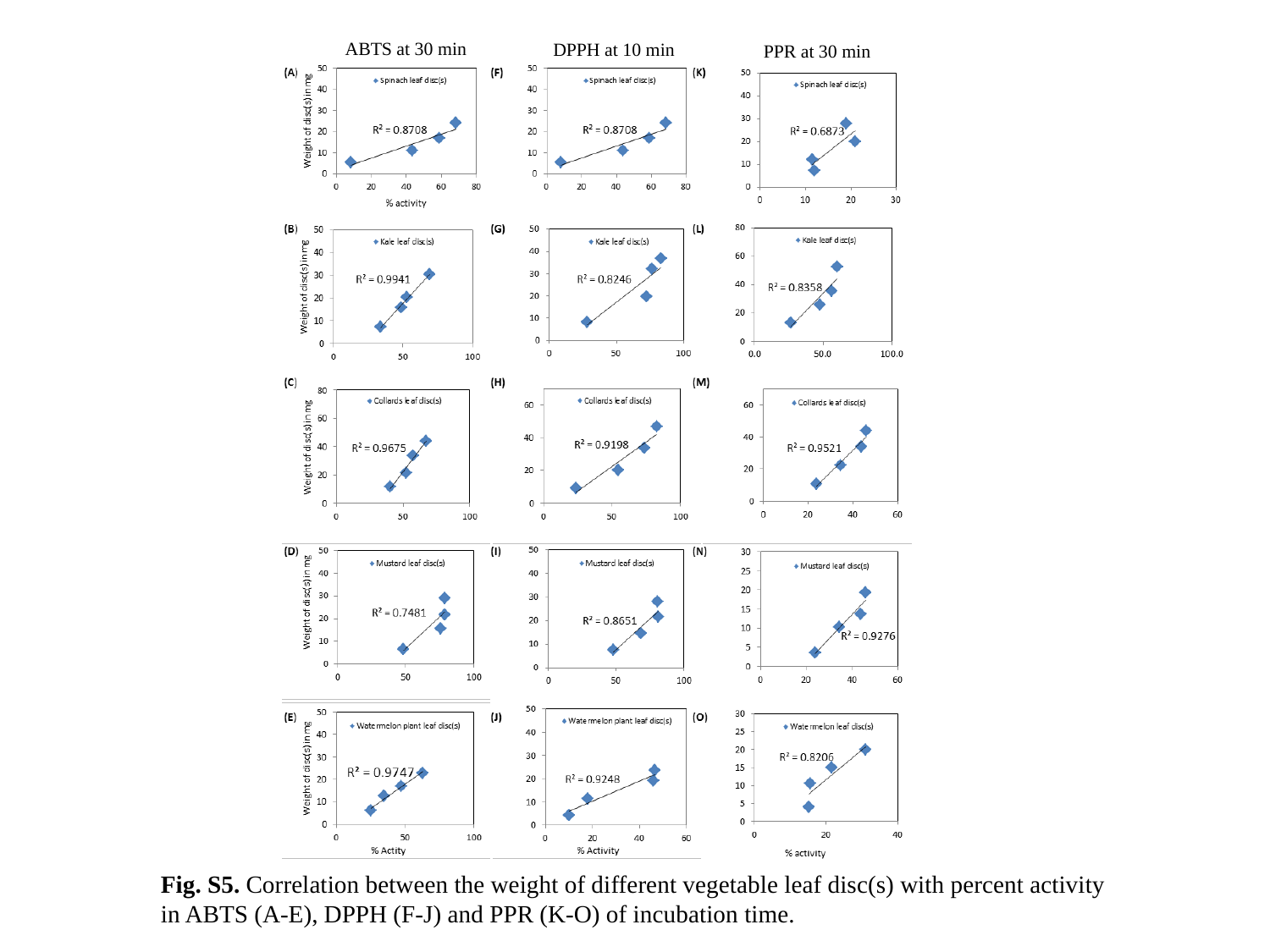

ABTS at 30 min
DPPH at 10 min
PPR at 30 min
Fig. S5. Correlation between the weight of different vegetable leaf disc(s) with percent activity in ABTS (A-E), DPPH (F-J) and PPR (K-O) of incubation time.

## Slide 7
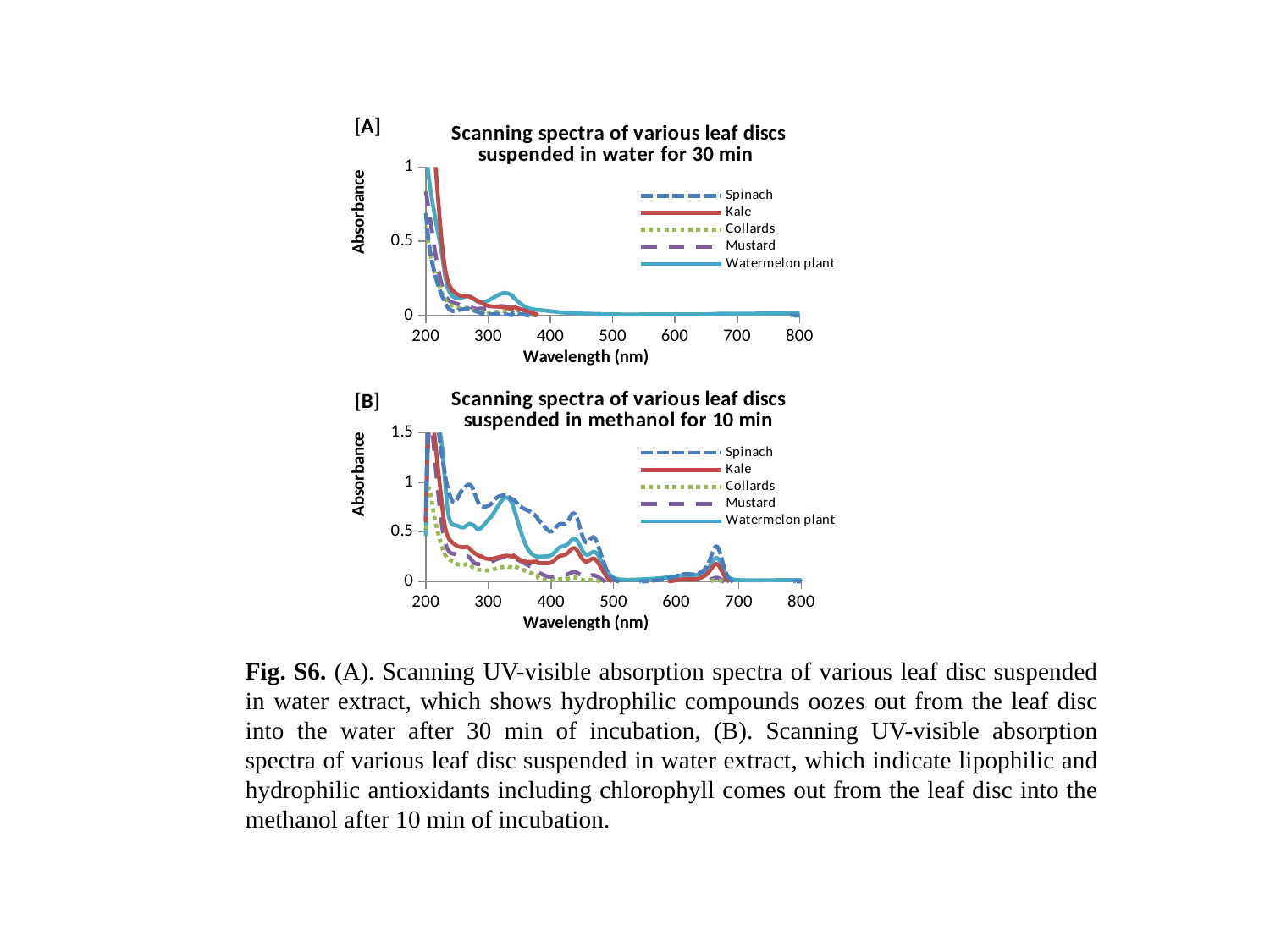

[A]
### Chart: Scanning spectra of various leaf discs suspended in water for 30 min
| Category | Spinach | Kale | Collards | Mustard | Watermelon plant |
|---|---|---|---|---|---|[B]
### Chart: Scanning spectra of various leaf discs suspended in methanol for 10 min
| Category | Spinach | Kale | Collards | Mustard | Watermelon plant |
|---|---|---|---|---|---|Fig. S6. (A). Scanning UV-visible absorption spectra of various leaf disc suspended in water extract, which shows hydrophilic compounds oozes out from the leaf disc into the water after 30 min of incubation, (B). Scanning UV-visible absorption spectra of various leaf disc suspended in water extract, which indicate lipophilic and hydrophilic antioxidants including chlorophyll comes out from the leaf disc into the methanol after 10 min of incubation.

## Slide 8
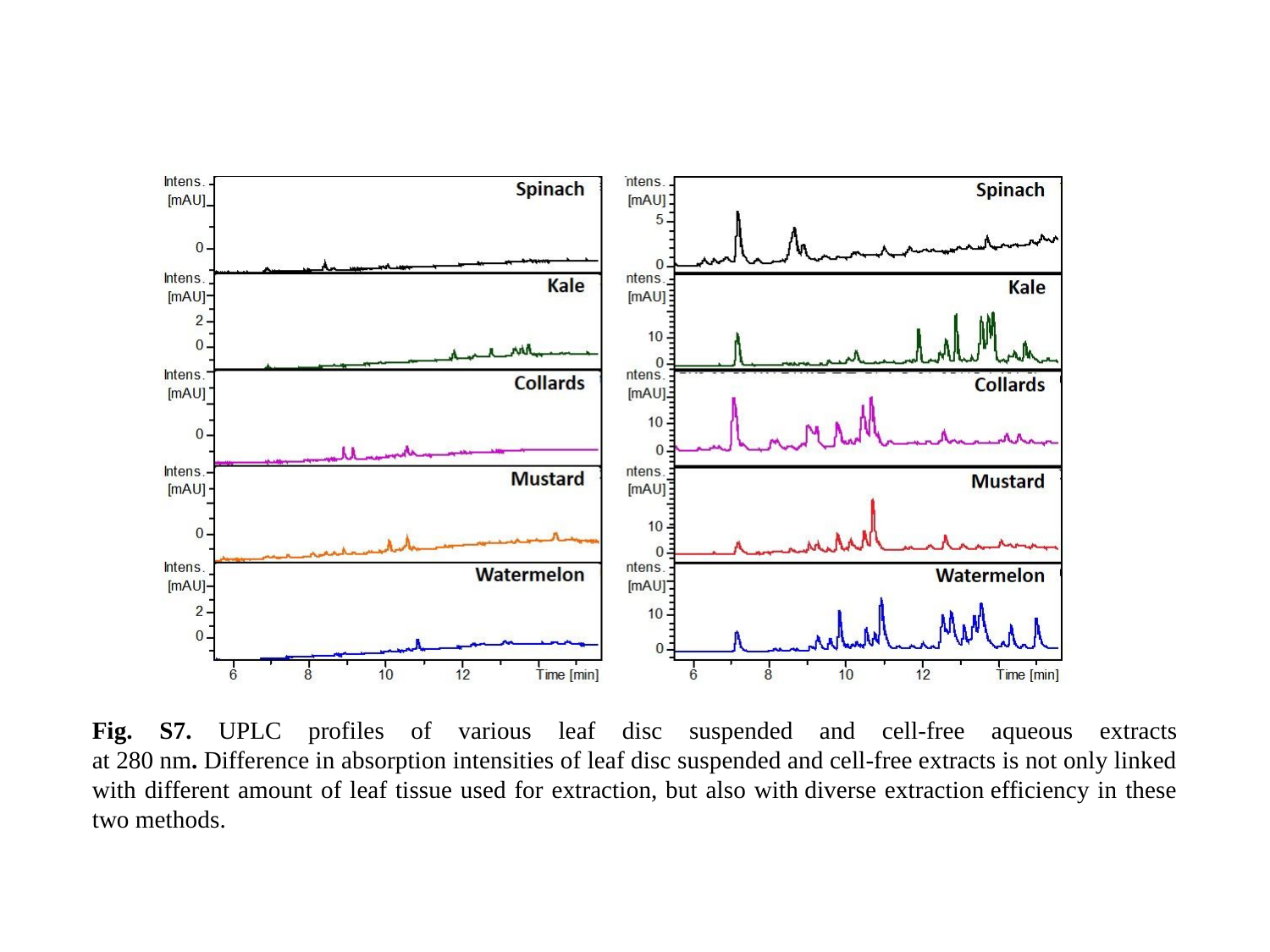

Fig. S7. UPLC profiles of various leaf disc suspended and cell-free aqueous extracts at 280 nm. Difference in absorption intensities of leaf disc suspended and cell-free extracts is not only linked with different amount of leaf tissue used for extraction, but also with diverse extraction efficiency in these two methods.

## Slide 9
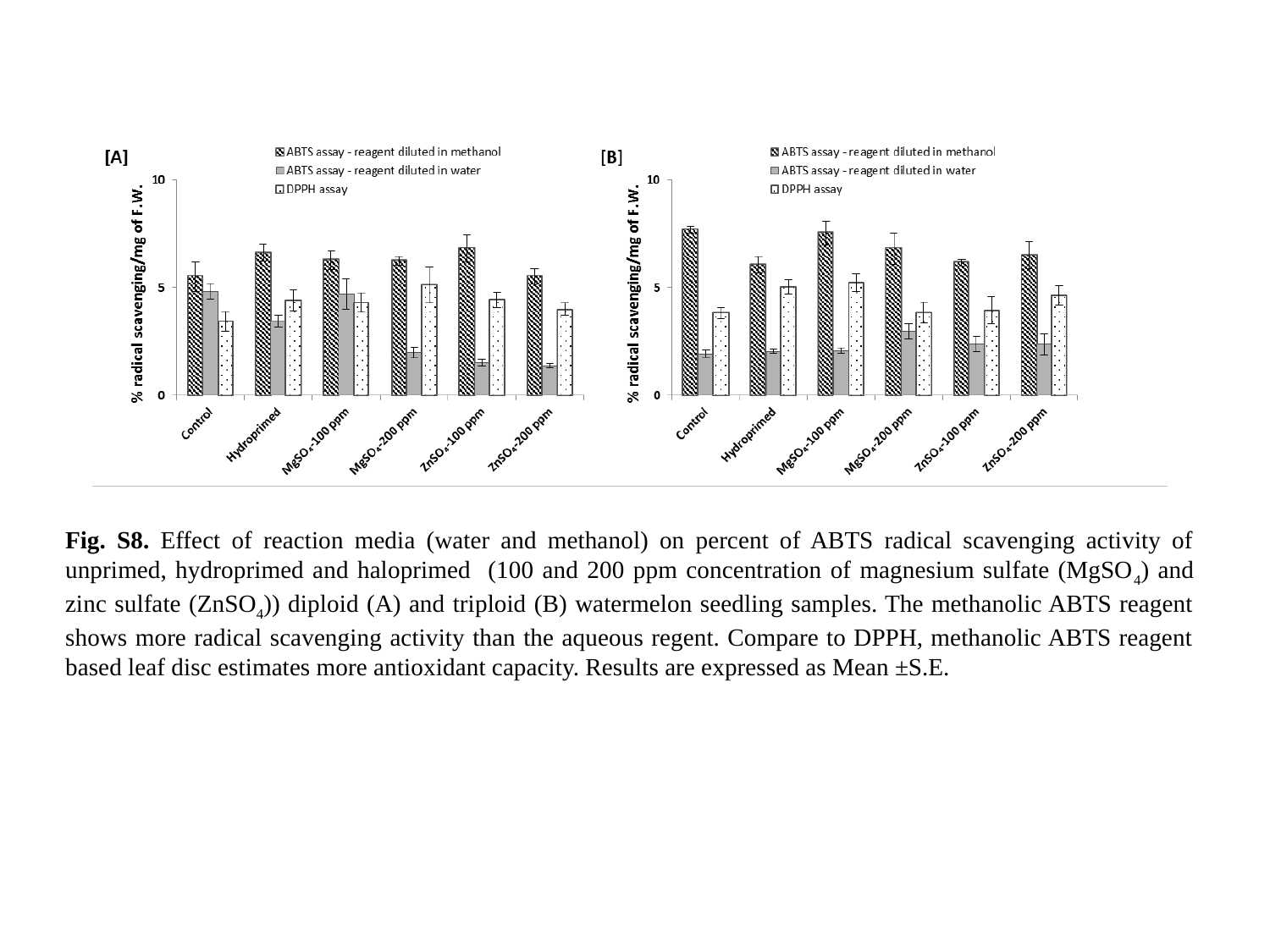

Fig. S8. Effect of reaction media (water and methanol) on percent of ABTS radical scavenging activity of unprimed, hydroprimed and haloprimed (100 and 200 ppm concentration of magnesium sulfate (MgSO4) and zinc sulfate (ZnSO4)) diploid (A) and triploid (B) watermelon seedling samples. The methanolic ABTS reagent shows more radical scavenging activity than the aqueous regent. Compare to DPPH, methanolic ABTS reagent based leaf disc estimates more antioxidant capacity. Results are expressed as Mean ±S.E.

## Slide 10
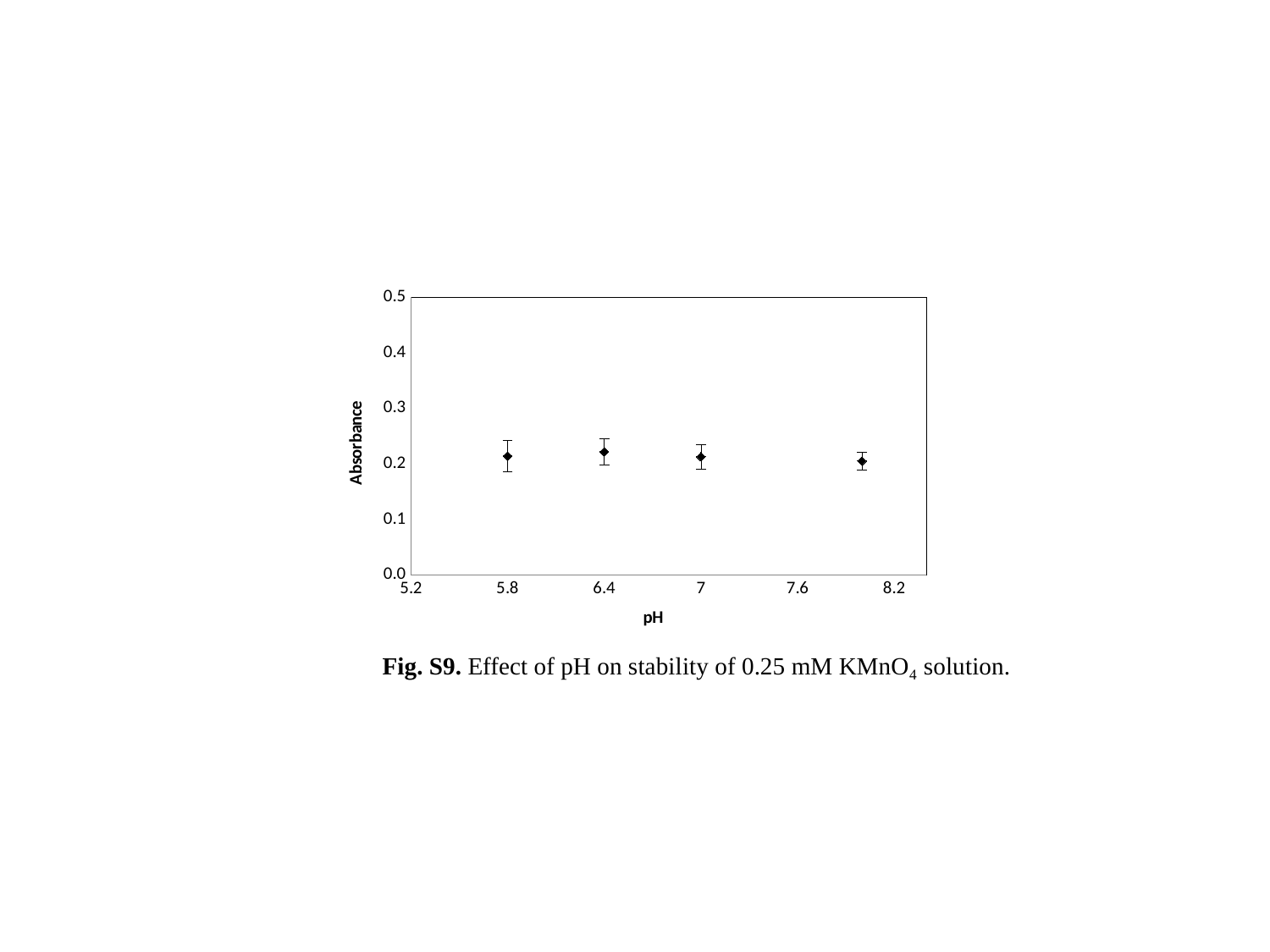

### Chart
| Category | |
|---|---|Fig. S9. Effect of pH on stability of 0.25 mM KMnO₄ solution.

## Slide 11
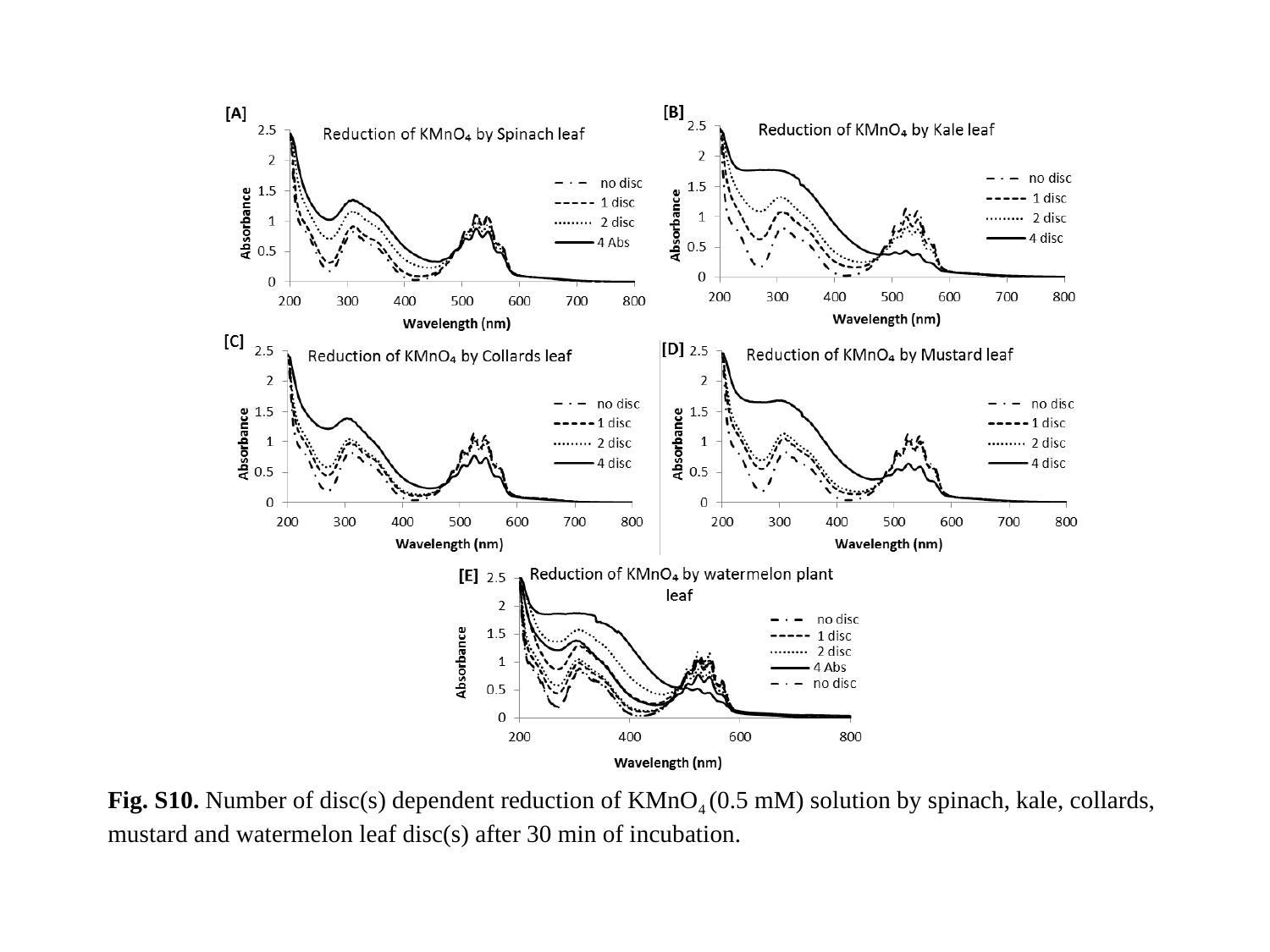

Fig. S10. Number of disc(s) dependent reduction of KMnO4 (0.5 mM) solution by spinach, kale, collards, mustard and watermelon leaf disc(s) after 30 min of incubation.

## Slide 12
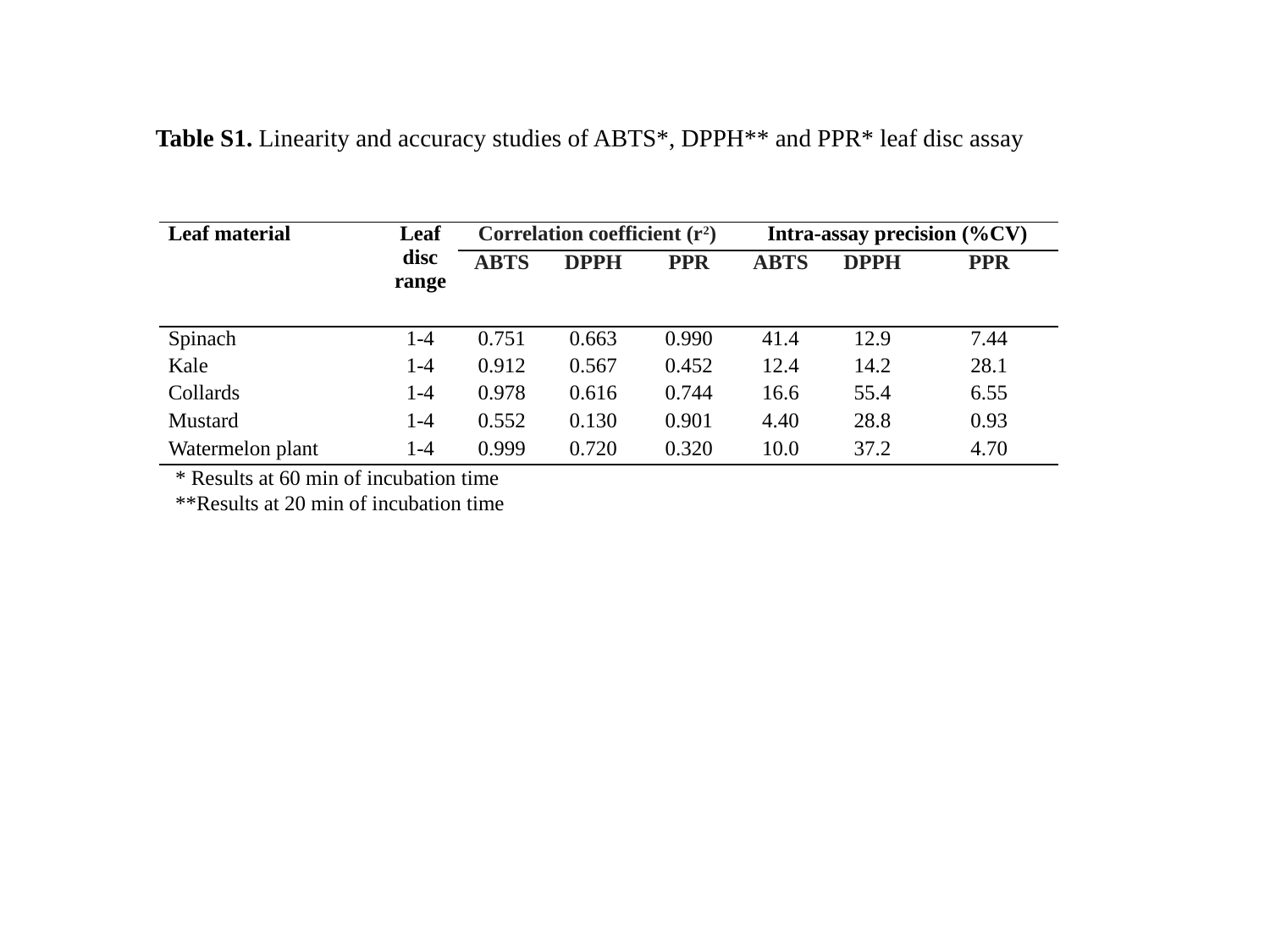

Table S1. Linearity and accuracy studies of ABTS*, DPPH** and PPR* leaf disc assay
| Leaf material | Leaf disc range | Correlation coefficient (r2) | | | Intra-assay precision (%CV) | | |
| --- | --- | --- | --- | --- | --- | --- | --- |
| | | ABTS | DPPH | PPR | ABTS | DPPH | PPR |
| Spinach | 1-4 | 0.751 | 0.663 | 0.990 | 41.4 | 12.9 | 7.44 |
| Kale | 1-4 | 0.912 | 0.567 | 0.452 | 12.4 | 14.2 | 28.1 |
| Collards | 1-4 | 0.978 | 0.616 | 0.744 | 16.6 | 55.4 | 6.55 |
| Mustard | 1-4 | 0.552 | 0.130 | 0.901 | 4.40 | 28.8 | 0.93 |
| Watermelon plant | 1-4 | 0.999 | 0.720 | 0.320 | 10.0 | 37.2 | 4.70 |
* Results at 60 min of incubation time
**Results at 20 min of incubation time

## Slide 13
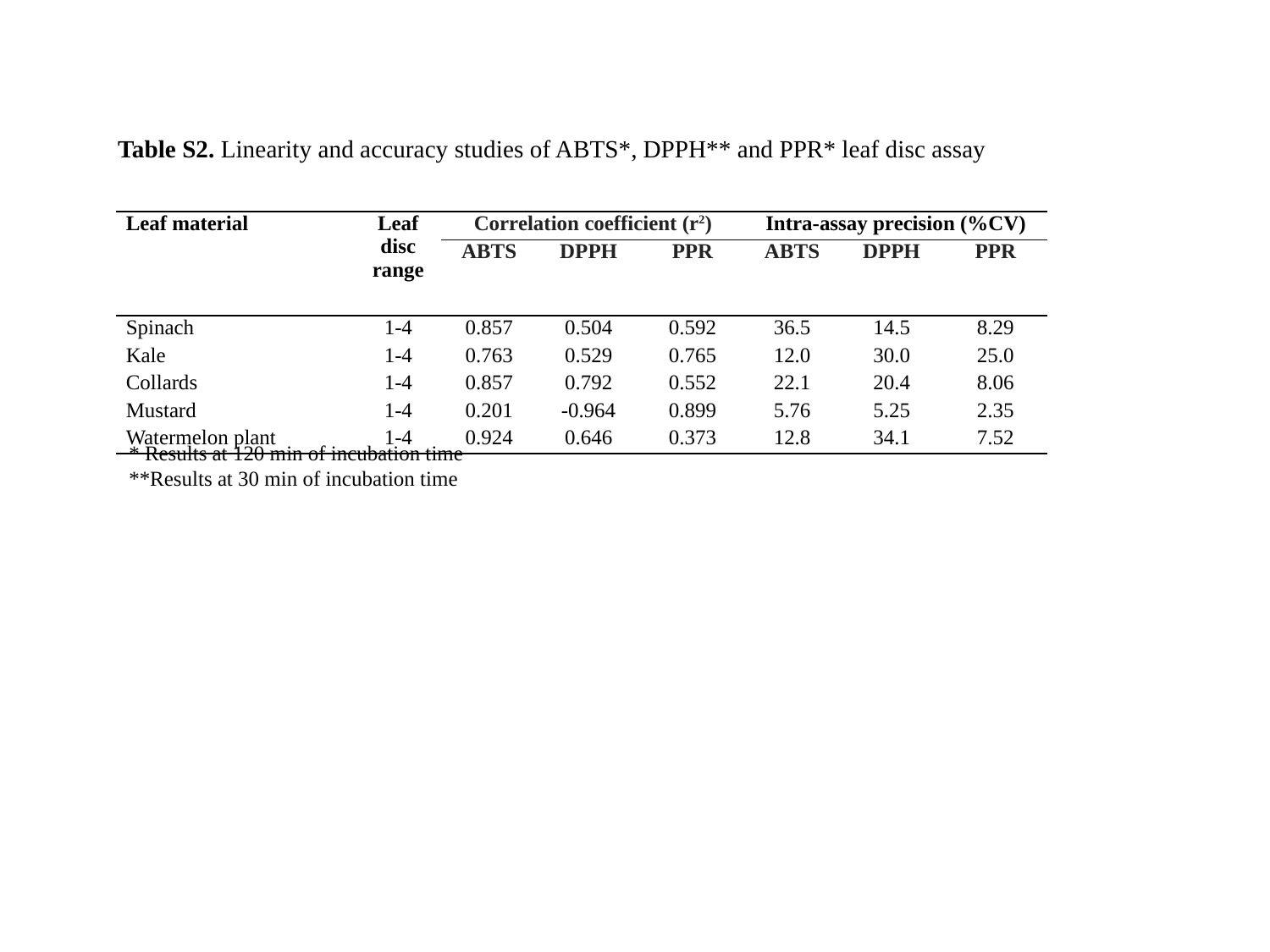

Table S2. Linearity and accuracy studies of ABTS*, DPPH** and PPR* leaf disc assay
| Leaf material | Leaf disc range | Correlation coefficient (r2) | | | Intra-assay precision (%CV) | | |
| --- | --- | --- | --- | --- | --- | --- | --- |
| | | ABTS | DPPH | PPR | ABTS | DPPH | PPR |
| Spinach | 1-4 | 0.857 | 0.504 | 0.592 | 36.5 | 14.5 | 8.29 |
| Kale | 1-4 | 0.763 | 0.529 | 0.765 | 12.0 | 30.0 | 25.0 |
| Collards | 1-4 | 0.857 | 0.792 | 0.552 | 22.1 | 20.4 | 8.06 |
| Mustard | 1-4 | 0.201 | -0.964 | 0.899 | 5.76 | 5.25 | 2.35 |
| Watermelon plant | 1-4 | 0.924 | 0.646 | 0.373 | 12.8 | 34.1 | 7.52 |
* Results at 120 min of incubation time
**Results at 30 min of incubation time
